# The prevalence of BRCA1 mutations in Chinese patients with early onset breast cancer and affected relatives

**DOI:** 10.1054/bjoc.1999.0960

**Published:** 2000-01-18

**Authors:** J-H Sng, J Chang, F Feroze, N Rahman, W Tan, S Lim, M Lehnert, S van der Pool, J Wong

**Affiliations:** Oncology Research Institute (National University Medical Institutes) and Department of MedicalOncology, National University of Singapore, Lower Kent Ridge Road, Singapore; Institute of Cancer Research, Downs Road, Sutton, UK

**Keywords:** BRCA1, Chinese, breast cancer

## Abstract

The purpose of this study was to determine the prevalence of BRCA1 mutations in Chinese breast cancer patients in Singapore. BRCA1 analysis was conducted in consecutive patients with breast cancer before the age of 40 years (76 women), or whose relatives had breast or ovarian cancer (16 women). Ten patients had both early onset breast cancer and affected relatives. Genomic DNA from peripheral mononuclear blood cells was studied by using the protein transcription–translation assay (exon 11) and single-strand conformational polymorphism, with subsequent DNA sequencing. All six disease-causing mutations occurred in women under 40 years (8.6%) with three occurring in patients under 35 years (three out of 22 patients, 13.6%). Mis-sense mutations of unknown significance were found in three patients. Two of the ten women with affected relatives under 40 years had BRCA1 mutations. The prevalence of BRCA1 mutations in Chinese patients with early onset breast cancer is similar to that observed in Caucasian women. Most Chinese patients with affected relatives were not carriers of BRCA1 mutations. © 2000 Cancer Research Campaign


*British Journal of Cancer* (2000) **82**(3), 538–542
© 2000 Cancer Research Campaign
DOI: 10.1054/ bjoc.1999.0960, available online at http://www.idealibrary.com on IDEAL®

### The prevalence of BRCA1 mutations in Chinese patients with early onset breast cancer and affected relatives

**J-H Sng[1,*], J Chang[1,*], F Feroze[1], N Rahman[2], W Tan[1], S Lim[1], M Lehnert[1], S van der Pool[1] and J Wong[1]**

[1]Oncology Research Institute (National University Medical Institutes) and Department of Medical Oncology, National University of Singapore, Lower Kent Ridge Road, Singapore; [2]Institute of Cancer Research, Downs Road, Sutton, UK

**Summary** The purpose of this study was to determine the prevalence of BRCA1 mutations in Chinese breast cancer patients in Singapore. BRCA1 analysis was conducted in consecutive patients with breast cancer before the age of 40 years (76 women), or whose relatives had breast or ovarian cancer (16 women). Ten patients had both early onset breast cancer and affected relatives. Genomic DNA from peripheral mononuclear blood cells was studied by using the protein transcription–translation assay (exon 11) and single-strand conformational polymorphism, with subsequent DNA sequencing. All six disease-causing mutations occurred in women under 40 years (8.6%) with three occurring in patients under 35 years (three out of 22 patients, 13.6%). Mis-sense mutations of unknown significance were found in three patients. Two of the ten women with affected relatives under 40 years had BRCA1 mutations. The prevalence of BRCA1 mutations in Chinese patients with early onset breast cancer is similar to that observed in Caucasian women. Most Chinese patients with affected relatives were not carriers of BRCA1 mutations. © 2000 Cancer Research Campaign

**Keywords**: BRCA1; Chinese; breast cancer

In Singapore, the incidence of breast cancer has doubled over the past two decades, with an annual increase significantly higher in premenopausal than post-menopausal women (5.7% vs 3.9%) (Seow et al, 1996). Of note, this annual increase is almost four-fold higher in premenopausal Singaporean Chinese women when compared to cancer incidence rates from Western countries (dos Santos Silva and Swerdlow, 1995). A hypothesis behind this observation may be a birth cohort effect associated with lifestyle changes like low parity and diet (Lee et al, 1991) as possible risk modifiers in women with a genetic predisposition to breast cancer (Narod et al, 1995; Chang-Claude et al, 1997).

In 1990, genetic linkage analysis of families with multiple cases of early breast cancer led to the localization of the breast cancer susceptibility gene BRCA1 to chromosome 17q (Hall et al, 1990) and subsequently to the cloning of this gene (Miki et al, 1994). Germline mutations of this gene appear to account for only about 40% of hereditary breast cancers and a minority of breast cancers with a family history (Couch et al, 1997). As of 1997, more than 350 sequence alterations have been listed which may be associated with breast cancer susceptibility (Breast Information Core, BIC). These mutations are scattered throughout the coding sequence of the gene, which is composed of 24 exons encoding for a 1863 amino acid protein. A majority are alterations that are predicted to affect the structure of the resulting proteins and thus are likely to be disease-causing mutations. In the case of BRCA1, approximately 40–50% of the alterations are frame-shift mutations, and 10–15% are non-sense mutations, both of which produce truncated proteins. Mis-sense mutations that are probably associated with increased risk of breast cancer account for 5–10% of the reported mutations, and the remaining 30–40% of the alterations are of unclear significance, either mis-sense or polymorphisms.

Mutations in BRCA1 are rare in the general Caucasian population, apart from the Ashkenazi Jews (Streuwing et al, 1995). Generally, results indicate about 5–6% of breast cancers under 40 years of age to be due to mutations in BRCA1 (Ford et al, 1995). To date, no systematic studies of BRCA1 mutations in Chinese patients with breast cancers have been reported. The objective of this study was to determine the prevalence of BRCA1 mutations in Chinese breast cancer patients in Singapore with early onset disease or a positive family history.

### METHODS AND MATERIALS

### Patients and determination of family history

From 1990 to 1998, 85 consecutive unrelated patients presenting to the National University Hospital were eligible for BRCA1 testing if they had a diagnosis of breast cancer before the age of 40 years (i.e. early onset), and/or at least one first-degree relative, or two second- or third-degree relatives with either breast or ovarian cancer.

Of all eligible patients, nine women declined genetic testing. Of the remaining 76 patients, 70 patients had early onset breast cancer, 16 patients had breast cancer affected relatives, while ten patients had both early onset breast cancer and affected relatives. This study had institutional ethical committee approval and signed written informed consent was obtained from each participant. The family histories on number and age of relatives, together with numbers of affected relatives and age of onset of cancers were

*Received 17 March 1999*
*Revised 13 July 1999*
*Accepted 23 July 1999*

*Correspondence to*: J Chang, Baylor Breast Care Center, 6560 Fannin, Suite 1558, Houston, TX 77030, USA

*Joint first authors.

determined by direct interviews by a single physician (JC). If BRCA1 mutations were identified, BRCA1 analysis was offered to first-degree female relatives of the patient.

### Molecular studies

### *DNA isolation*

Blood samples were obtained for the extraction of genomic DNA, which was isolated from peripheral blood mononuclear cells using standard procedures (Sambrook et al, 1989).

### *Single-strand conformation polymorphism and DNA sequence analysis*

Single-strand conformation polymorphism (SSCP) analysis using primer pairs that span the BRCA1 coding region (BIC database) and intron–exon boundaries was performed for all coding exons except for exon 11. Polymerase chain reaction (PCR) amplification of genomic DNA was carried out in 10-μl volumes containing 50 ng of genomic DNA, 1.5 mM magnesium chloride, 50 mM potassium chloride, 10 mM Tris–HCl (pH 8.3), 200 μM dNTPs (Promega, USA), 0.8 μM of each primer and 0.75 units of *Taq* polymerase (Promega, USA). Amplification was for 35 cycles in a Perkin-Elmer 480 DNA thermal cycler (30 s at 94°C, 1 min at the respective annealing temperature, and 1 min at 72°C, with a 10-min extension at 72°C after the last cycle). A 1.2-μl aliquot of the PCR product was diluted into 4.9 μl of denaturing loading buffer (95% formamide, 10 mM sodium hydroxide, 0.05% xylene cyanol FF and 0.05% bromophenol blue), heated at 94°C for 5 min, cooled on ice for 5 min and loaded for electrophoresis. SSCP gels consisting of 0.5× Mutation Detection Enhancement solution (FMC Bioproducts) in 0.6× Tris–borate EDTA (TBE) buffer were run in 0.6× TBE buffer at 4 W for 18–22 h at 4°C. After electrophoresis, the SSCP gels were silver-stained and the DNA bands of variant and wild-type mobility were excised from the gels and eluted into 50 μl of TE (pH 8.0) by incubating at 37°C for 2 h. The eluted DNA (10 μl) was used as a template for subsequent PCR amplification. The PCR products were purified using the Wizard PCR Prep DNA Purification System (Promega, USA) and double-stranded sequencing was performed using the Sequenase PCR Product Sequencing Kit (United States Biochemical, USA).

After completion of the sequencing reaction, the samples were denatured at 82°C for 2–3 min, and 2.5 μl were loaded onto a polyacrylamide gel containing 7M urea. Electrophoresis was at 60–70 W for 4 h at room temperature. Sequencing gels were fixed with 10% methanol–10% glacial acetic acid, dried and exposed to autoradiography overnight at room temperature. If mutations were detected, a second SSCP-sequencing analysis was performed for confirmation.

### *Protein transcription–translation assay*

Protein transcription–translation (PTT) analysis was used to detect truncating mutations in exon 11 (Cornelisse et al, 1995; Hogervorst et al, 1996). Exon 11 was amplified in three overlapping fragments, ranging in size from 1275 to 1600 bp, using previously published primers (Plummer et al, 1995). The 5′ oligonucleotide for each of the three PCR fragments contained a T7 polymerase recognition site, a Kozak consensus sequence and a start codon to allow the transcription and translation of uncloned PCR products. PCR was performed in 50 μl volumes, containing 1× PCR reaction buffer, 0.2 μM of each dNTP, 0.8 μM of each primer, 0.75 units of *Taq* polymerase (Promega, USA) and 50 ng of template DNA. The reactions were amplified under the following conditions: 1 cycle at 94°C for 3 min; 35 cycles at 94°C for 30 s; 52–58°C for 30 s and 72°C for 1 min with the final extension at 72°C for 10 min. PCR products were purified, and the mRNA was translated into radio-labelled peptides using the TnT™ T7 Coupled Reticulocyte Lysate System or Wheat Germ System (Promega, USA). [$^{35}$S]-methionine/cysteine (NEN Research Products, USA) was used for radioactive labelling of the translation products. The products were size separated on 12% sodium dodecyl sulphate-polyacrylamide (SDS-PAGE) gels. After electrophoreses, the gels were dried and autoradiographed on Hyperfilm™ (Amersham, UK) overnight at –70°C. If truncations were detected, DNA sequencing was performed as described above.

**Table 1** BRCA1 mutations in patients with a positive family history

| Patient no. | Age (years) | Family history of cancer (age, years) | Mutations |
|---|---|---|---|
| 1 | 33 | Mother: breast (50) | **C→T at nt4446** |
| | | Maternal grandmother: breast (50) | |
| | | Maternal aunt: breast (46) | |
| 2 | 39 | Maternal aunt: breast (60s) | **2732insT** |
| | | Paternal cousin: breast (50s) | |
| | | Father: lung (70) | |
| | | Paternal cousin: brain (60) | |
| 3 | 32 | Sister: breast (28) | Not detected |
| | | Paternal cousin: breast (37) | |
| 4 | | Maternal grandmother: breast (50) | Not detected |
| | 39 | Paternal aunt: breast (60) | |
| | | Paternal aunt: ovarian (70) | |
| 5 | 39 | Sister: breast (39) | Not detected |
| | | Paternal cousin: bilateral breast (39) | |
| 6 | 39 | Sister: breast (40) | Not detected |
| 7 | | Maternal cousin: breast (40) | Not detected |
| | 36 | Maternal cousin: ovarian (40) | |
| | | Maternal cousin: ovarian (40) | |
| 8 | 39 | Sister: breast (43) | Not detected |
| 9 | 39 | Sister: breast (40) | Not detected |
| 10 | 34 | Mother: breast (44) | Not detected |
| 11 | 55 | Sister: breast (50) | Not detected |
| | | Niece: breast (50) | |
| 12 | 53 | Daughter: breast (36) | Not detected |
| 13 | 52 | Maternal grandmother: breast (70) | **Val191Ile** |
| | | Maternal aunt: breast (50) | |
| | | Maternal aunt: ovarian (35) | |
| 14 | 48 | Mother: breast (50s) | Not detected |
| 15 | 52 | Sister: breast (40) | Not detected |
| 16 | 40 | Mother: breast (40) | Not detected |
| | | Paternal cousin: bilateral (NK) | |

### RESULTS

### Patients' characteristics

A total of 76 Chinese patients with breast cancer were analysed for BRCA1 germline mutations. Seventy patients had breast cancer under the age of 40 years, and 22 had breast cancer under the age of 35 years. Sixteen patients had affected relatives, ten with breast cancer under the age of 40 years and 6 at 40 years and above respectively. The family histories of these women are summarized in Table 1.

*© 2000 Cancer Research Campaign*

### Characterization of alterations in BRCA1 sequence

*Prevalence*

In the 70 patients with breast cancer under the age of 40 years, six disease-causing mutations (8.6%) and three mis-sense mutations of unknown significance were detected. In the 22 patients with cancer under the age of 35 years, three disease-causing mutations (13.6%) were found.

In those with affected relatives, two patients with breast cancer before the age of 40 had disease-causing mutations, while a mis-sense mutation was noted in a patient with breast cancer diagnosed at 52 years (Table 1).

*Disease-causing BRCA1 mutations*

Of the six disease-causing mutations two occurred in patients (BSF and MN) with early onset breast cancer and a positive family history. The mutation found in patient BSF had not been published previously and consisted of insertion of T at nt2732, resulting in chain termination at codon 902 (Figure 1). The mutation in patient MN was a non-sense mutation with a C to T substitution at nt4446, resulting in a termination codon (Figure 2). The mother of MN who had breast cancer, and the sister who had no history of cancer, were found to harbour the same C→T mutation at nt4446. This particular mutation has been previously described in 14 different families including one family with 11 breast and ovarian cancers (Couch et al, 1996).

*Mis-sense mutations of unknown significance*

An A→G mutation at nt3667 (Lys1183Arg) was detected in two patients. One of them (TJG) also had a disease-causing mutation (3378/3381delG). The other patient (YW) had an additional mis-sense mutation at nt3300 (T→A, Ser 1040Thr).

A rare missense mutation was found in two other women (TGN, TPC) with a G→A substitution (Val191Ile) in exon 9. Patient TGN had breast cancer at 52 years and a positive family history, while TPC had early onset breast cancer.

*Polymorphisms*

Twelve polymorphisms were identified representing sequence changes in exon 3 (eight women at nt38, G→A mutation, Lys → Lys), in exon 13 (three women at nt1436, T→C mutation, Ser → Ser) and intron 18 (one woman at nt5272/5273, G→A mutation).

### DISCUSSION

The prevalence of BRCA1 germline mutations in Singaporean Chinese with early onset breast cancer under the age of 35 and 40 years were approximately 14% and 9% respectively. This is similar to Western series, apart from the Ashkenazi Jews. In a UK population-based study of 640 women, 3% of patients diagnosed under 35 years and 5.3% of patients diagnosed under 40 years were carriers of BRCA1 mutations (Ford et al, 1989). In a US study, 7.5% of patients (six out of 80) under the age of 35 were carriers of BRCA1 mutations (Langston et al, 1996). In a later update of this series, 6.2% of patients under 35 years and 7.2% of patients under 45 years with a first-degree family history were found to be carriers of BRCA1 mutations (Malone et al, 1998). Data from several authors suggest a limited role of BRCA1 mutations in early onset breast cancer among Japanese patients (Inou et al, 1995; Katagiri et al, 1996). The highest prevalence of BRCA1 mutations has been found in Ashkenazi Jewish women, with approximately 20% of breast cancer patients under the age of 40 carrying the BRCA1 185delAG mutation (Streuwing et al, 1995).

In the present series of 16 Chinese patients with affected relatives, disease-causing BRCA1 mutations were found in two patients who were diagnosed under the age of 40 years. Singapore had practised a 2-child policy from the 1960s to the 1980s which has limited the number of large family pedigrees. Our data suggest that Singaporean Chinese patients with breast cancer and affected relatives have a low probability of carrying germline BRCA1 mutations. This suggests that mutations in other cancer predisposition genes such as BRCA2, ataxia-telangiectasia gene (*ATM*) or *TP53* tumour suppressor gene may play a role in familial breast cancers in Singaporean Chinese.

In our population, mutations were found mainly in exon 11 which represents 60% of the gene. The non-sense mutation C→T at nt4446 of exon 13 was found in a patient and two first-degree female relatives. The same mutation has been previously detected in 14 different families (Couch et al, 1996). So far, studies with polymorphic marker haplotypes within or close to BRCA1 have suggested that almost all recurrent familial mutations in BRCA1 originate from a common founder (Neuhasen et al, 1996). However, our observation of this C→T at nt4446 mutation in a Chinese family suggests that this mutation may rather represent a 'hot spot' for mutagenesis.

**Table 2** BRCA1 mutations in patients with early onset breast cancer

| Initials | Age (years) | Exon | Codon | Nucleotide change | Consequence | Affected relatives |
|---|---|---|---|---|---|---|
| Disease-causing mutations | | | | | | |
| TSL | 35 | 11A | 464 | 1510delC | Truncation at codon 474 | N |
| WMC | 37 | 11A | 468 | 1523delG | Truncation at codon 475 | N |
| YSG | 34 | 11B | 770 | 2430insC | Truncation at codon 776 | N |
| TJG | 39 | 11C | 1088 | 3378/81delG | Truncation at codon 1108 | N |
| BSF | 39 | 11B | 871 | 2732insT | Truncation at codon 902 | Y |
| MN | 33 | 13 | 1443 | 4446C→T | Truncation at codon 1443 | Y |
| Unclassified variants | | | | | | |
| TJG | 39 | 11C | 1183 | 3667A→G | Lys1183Arg | N |
| YW | 39 | 11C | 1183 | 3667A→G | Lys1183Arg | N |
| | | | 1040 | 3300T→A | Ser1040Thr | |
| TPC | 38 | 9 | 119 | 690G→A | Val191Ile | N |

del, deletion; ins, insertion.

*© 2000 Cancer Research Campaign*

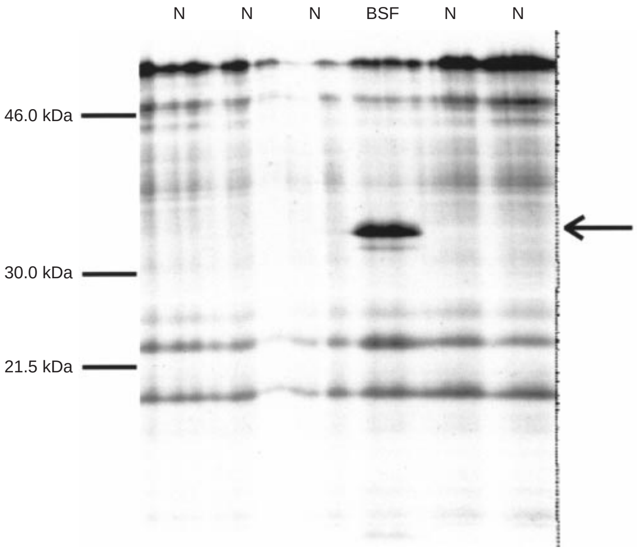

**Figure 1** Mutational analysis of BRCA1 exon 11 using PTT. The panel shows the electrophoretic analysis of peptides generated by the PTT assay. The lane of patient BSF shows a truncated protein (arrowed), and the full-length normal product. DNA sequencing revealed premature stop codon at 902 (mutation, 2732insT) in one BRCA1 allele. Bands from five other patients show normal (N) electrophoretic band

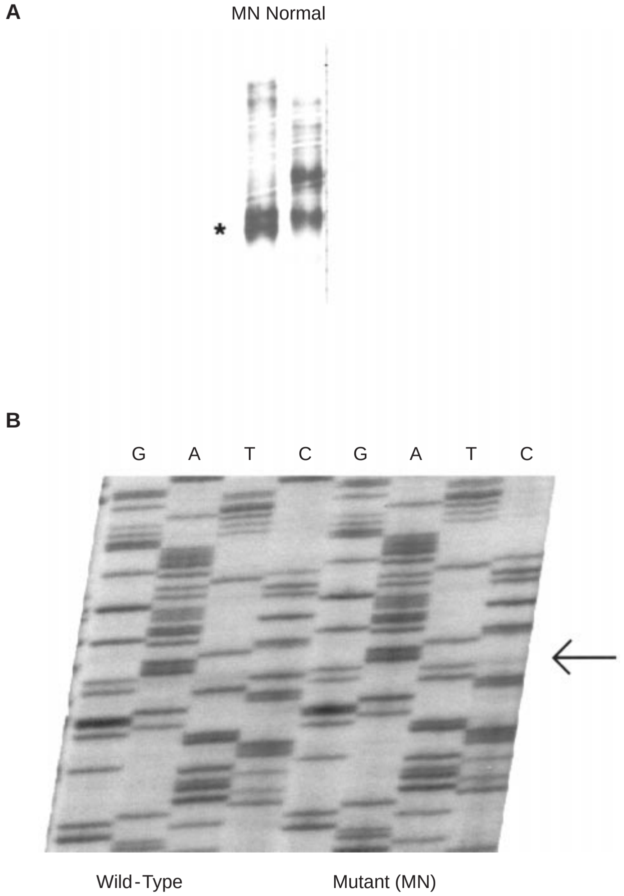

**Figure 2** BRCA1 mutation identified by SSCP analysis and sequencing. The autoradiograph of a SSCP gel (**A**) shows the normal migration pattern of a denatured PCR product (Normal) and the mobility shift (*) produced by a nonsense mutation in exon 13 (patient MN). Sequence analysis (**B**) revealed a C→T substitution (arrowed) at codon 1443, changing the wild-type CGA to TGA

A mis-sense mutation in exon 9 (Val191Ile) has now been found in three Chinese women with breast cancer, two in this series and one in a Taiwanese Chinese woman with breast cancer at 34 years (D Shattuck-Eidens, personal communication). Distinguishing between disease-causing mis-sense mutations and rare polymorphisms remains problematic. Substitutions that occur in highly conserved regions like the ring-finger domain, show segregation with disease in high-risk families, and are not observed in controls are typically classified as pathogenic. The mis-sense mutation Val191Ile is located close to the ring-finger binding domain and highly conserved as compared to murine BRCA1 (Sharan et al, 1995). Unfortunately, investigation of segregation of this mutation with disease in our family with multiple cancers (TGN) was unsuccessful as her relatives declined investigation.

Another unclassified variant Lys1183Arg was identified in two women, of whom one had a definite truncated peptide at codon 1108. As such, this alteration is more likely to represent a polymorphism. Alternatively, Lys1183Arg may be present in linkage disequilibrium with a definite disease-causing mutation (Dunning et al, 1997). Finally, the polymorphisms observed in this series have been noted by other investigators in control subjects and patients (BIC database), and are unlikely to be associated with the penetrant phenotypes normally seen in families with disease linked to the BRCA1 region.

This is the first systematic study on BRCA1 mutations in a large series of Chinese women with early onset and familial breast cancer. The prevalence of mutations in patients with early onset breast cancer is similar to that observed in Caucasian women. Only few patients with a positive family history were carriers of BRCA1 mutations. Studies are in progress which will determine the prevalence of other cancer predisposition genes and the effect of BRCA1 mutations on tumour phenotype and prognosis.

### ACKNOWLEDGEMENTS

We would like to thank Dr A Seow from the Department of Occupational and Family Medicine, National University of Singapore for her input on cancer trends in Singapore, and the National Medical Research Council for financial support (grant no: RP6600007). Presented in part at the 23rd European Society of Medical Oncology Congress in November 1998, Athens, Greece.

### REFERENCES

Breast Information Core at www.nhgri.nih.gov/Intramural_research/Lab_transfer/Bic

Chang-Claude J, Becher H, Eby N, Bastert G, Wahrendorf J and Hamann U (1997) Modifying effect of reproductive risk factors on age at onset of breast cancer for German BRCA1 mutation carriers. *J Cancer Res Clin Oncol* **123**: 272–279

Cornelisse CJ, den Dunnen JT, Devilee P and van Ommen G-JB (1995) Rapid detection of BRCA1 mutations by the protein truncation test. *Nat Genet* **10:** 208–212

Couch FJ, Weber BL and Breast Information Core (1996) Mutations and polymorphisms in the familial early-onset breast cancer (BRCA1) gene. *Hum Mutat* **8**: 8–18

dos Santos Silva I and Swerdlow AJ (1995) Recent trends in incidence of and mortality from breast, ovarian and endometrial cancers in England and Wales and their relation to changing fertility and oral contraceptive use. *Br J Cancer* **72**: 485–492

*© 2000 Cancer Research Campaign*

Dunning AM, Chiano M, Smith NR, Dearden J, Gore M and Oakes S (1997) Common BRCA1 variants and susceptibility to breast and ovarian cancer in the general population. *Hum Mol Genet* **6**: 285–289

Ford D, Easton DF and Peto R (1995) Estimates of the gene frequency of BRCA1 and its contribution to cancer incidence. *Am J Cancer Genetics* **57**: 1457–1462

Hall JM, Lee MK, Newman B, Morrow JE, Anderson LA, Huey B and King MC (1990) Linkage of early-onset familial breast cancer to chromosome 17q21. *Science* **250**: 1684–1689

Hogervorst FBL, Cornelis RS, Bout M, van Vliet M, Oosterwijk JC, Olmer R, Bakker B, Klijn JGN, Vaser HFA, Meijers-Heijboer H and Menko F (1996) Human, canine and murine BRCA1 genes: sequence comparison among species. *Hum Mol Genet* **5(9)**: 1289–1298

Inou R, Fukutomi T, Ushijima T, Matsumoto Y, Sugimura T and Naga M (1995) Germline mutation of BRCA1 in Japanese breast cancer families. *Cancer Res* **15**: 3521–3524

Katagiri T, Emi M, Ito I, Kobayashi K, Yoshimoto M, Iwase T, Kasumi F, Miki Y, Skolnick MH and Nakamura Y (1996) Mutations in the BRCA1 gene in Japanese breast cancer patients. *Hum Mutat* **7**: 334–339

Langston AA, Malone KE, Thompson JD, Daling JR and Ostrander EA (1996) BRCA1 mutations in a population-based sample of young women with breast cancer. *N Engl J Med* **334**: 137–142

Lee HP, Gourley L and Duffy SW (1991) Dietary effects on breast-cancer risk. *Lancet* **337**: 1197–1200

Malone KE, Daling JR, Thompson JD, O'Brien CA, Francisco LV and Ostrander EA (1998) BRCA1 mutations and breast cancer in the general population: analyses in women before age 35 years and in women before 45 years with first-degree family history. *JAMA* **279**: 922–929

Miki Y, Swensen J and Shattuck-Eidens D (1994) A strong candidate for the breast and ovarian cancer susceptibility gene BRCA1. *Science* **266**: 66–71

Narod SA, Goldgar D, Cannon-Albright L, Weber B, Molslehi R, Ives E, Lenoir G and Lynch H (1995. Risk modifiers in carriers of BRCA1 mutations. *Int J Cancer* **64**: 394–398

Neuhasen SL, Mazoyer S, Friedman L, Stratton M, Offitt K, Calligo A, Tomlinson G and Cannon-Albright Ll (1996) Haplotype and phenotype analysis of six recurrent BRCA1 mutations in 61 families: results of an international study. *Am J Hum Genet* **58**: 271–280

Plummer SJ, Anton-Culver H, Webster L, Noble B, Liao S, Kennedy A, Belinson J and Casey G (1995) Detection of BRCA1 mutations by the protein truncation test. *Hum Mol Genet* **4**: 1989–1991

Sambrook J, Fritsch EF and Manitas T (1989) *Isolation of High Molecular Weight DNA from Mammalian Cells.* Cold Spring Harbor Laboratory Press: New York, pp. 14–23

Seow A, Duffy SW, McGee MA and Lee HP (1996) Breast cancer in Singapore: trends in incidence 1968–1992. *Int J Epid* **25**: 40–45

Sharan SK, Wims M and Bradley A (1995) Murine BRCA1: sequence and significance for human mis-sense mutation. *Hum Mol Genet* **4**: 2275–2278

Streuwing JP, Abeliovich D, Peretz T, Avishai N, Kaback M, Collins FS and Brody LC (1995) The carrier frequency of BRCA1 185delag is approximately 1 percent in Ashkenazi Jewish individuals. *Nat Genet* **11**: 198–200

*© 2000 Cancer Research Campaign*
